# Supplementary material for: Evaluating clinician acceptability of the prototype CanRisk tool for predicting risk of breast and ovarian cancer: A multi-methods study
Source: PLoS One. 2020 Mar 6;15(3):e0229999. doi: 10.1371/journal.pone.0229999 (PMC7059924; doi:10.1371/journal.pone.0229999)
Supplement: S1 File — (DOCX) [file pone.0229999.s001.docx]

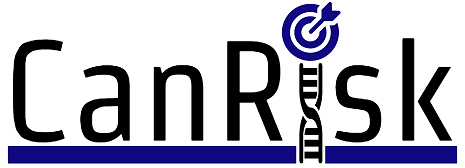


**CanRiskTool Evaluation Phase I**

**Interview Schedule**

**­**

**Introduction**

Thank you for doing the simulated consultations. I would now like to ask you a few questions around the experience of using the CanRisk tool. A reminder that this interview will be tape recorded.

**Questions:**

1. What do you think of the CanRisk tool in general?

*Prompt: Did you like it? Or not like it? Why/Why not?*

*Time dependant: can we walk through each section together? (Use screen shots)*

1. Did you find it easy to use in the consultations?

*Prompt: What made it easy to use? And less easy to use? Usefulness, functionality etc.*

1. What do you think about the look of the Can Risk tool?

*Prompt: Do you like the colours? And the graphics?*

1. How did you find the text/questions? Were they all easy to understand?

*Prompt: Were any area less easy to understand? Printed, laminated Screenshots of CanRisk tool*

1. What did you think of the family history section?

*Prompt: How do you normally record Family History? Was it intuitive? Easy to understand and complete?*

1. What did you think about the way the risk was presented?

*Prompt: Did it help in communicating the risk? Did you agree with the risk assessment? Why?*

1. How did the CanRisk tool impact on your interaction with the ‘patient’ during the consultation?

*From field notes can play back sections of the recording and ask what the specific issue was “what was going on here?” Prompt: comfort, interaction between doctor and patient maintained, patient centred, ease of communication, effect on patient*

1. Would you make any improvements to the CanRisk tool?

*Prompt: What could help it to work more effectively in your healthcare setting?*

1. Are there components that should not be altered? Which ones?

*Prompt: What did we get right?*

1. Might the CanRisk tool be useful in your consultations?

*Prompt: would you trust the information generated by the tool?*

1. Have you any concerns about the regulation and certification of electronic decision support such as the CanRisk tool?
2. Do you have any other comments on the CanRisk tool or cancer risk prediction?
3. Would you be willing to take part in future evaluations of the revised CanRisk tool?
