# Supplementary material for: Evaluating clinician acceptability of the prototype CanRisk tool for predicting risk of breast and ovarian cancer: A multi-methods study
Source: PLoS One. 2020 Mar 6;15(3):e0229999. doi: 10.1371/journal.pone.0229999 (PMC7059924; doi:10.1371/journal.pone.0229999)
Supplement: Attachment — Submitted filename: Response to reviewer comments Final.docx [file pone.0229999.s002.docx]

We would like to thank the reviewers and editorial staff providing insightful feedback on our manuscript. We have made the requested changes, and believe the manuscript is much stronger as a result. We have included a detailed response to each point in the document below, and where possible we have included the new manuscript text, marked in red.

**Reviewer 1 comments**

**1 The authors should report a kappa on the concordance of the coding of the transcripts between the two coders.**

Thank you for your suggestion. Two authors discussed the consistency of coding throughout the coding process, but formal analysis of the concordance between the coders was not performed. We have included additional detail in the analysis section of the method to reflect this (see page 7):

*In order to become familiar with the data, two authors (SA and FS) repeatedly read the interview transcripts, and notes were made about potential codes; these were subject to ongoing discussion and refinement. Following this, the same authors performed formal coding using NVivo [19], working through the transcripts and writing notes to support data patterns – these patterns were extracted as codes. Consistency of coding between SA and FS was discussed regularly through team meetings, but formal analysis of the concordance between reviewers was not completed.*

**2 In the discussion, the authors described barriers to the use of the tool in the primary care setting largely because of competing demands and time constraints in clinical practice. The authors should acknowledge the limitations of using this tool in a busy primary care practice.**

Thank you for including this suggestion. We have included this point in the opening section of the discussion (page 17) which now reads:

*The foremost concern for clinicians from the primary care setting was the amount of time needed to complete and interpret a risk calculation, highlighting a potential barrier to the use of the CanRisk tool in this setting.*

**3 In future studies, the authors could consider evaluating the patient experience in using this tool, as patient participation is also vital to the success of such an intervention. There were concerns raised by practitioners using the tool regarding insensitive and intrusive questions (for example, regarding stillbirths and miscarriages); patient feedback in this area would be particularly relevant. The authors also discuss potentially using family history intake via the patient portal and patient feedback would also be helpful in creating this.**

Thanks for raising this important point. We have edited the text in the ‘next steps’ section (see page 18) to reflect this:

*Further development and testing of the CanRisk tool is required. The findings presented here have already underpinned a number of changes to the prototype CanRisk tool (see Box 3). Whilst these changes may go some way to improving the tool for clinical users in a variety of settings, further research exploring the patient experience of the tool and its use in clinical practice is necessary. Data collected in these studies would allow us to understand the acceptability of the CanRisk tool in this important stakeholder group and may inform future patient centred design.*

4 Another potential challenge to the implementation of this tool raised by authors is the interpretation and communication of risk estimates to patients by primary providers (not the genetics specialists). This is clearly an unmet need in primary care, and education regarding communicating cancer risk and options for risk management are clearly required for this tool to be useful in a generalist practice.

Thank you for raising this point. We have now edited the text (see page 18) to further emphasise the need for the development and testing of learning resources prior to implementation:

*Additional learning resources focusing on the communication of multifactorial risk for those in the specialist genetics setting and genetic risk for those in the primary care setting may also support clinicians to communicate the results more effectively. The development and testing of these resources should be considered prior to implementation.*

**Reviewer 2 comments**

**1 Abstract: The summary of the results in the abstract is quite vague and doesn’t highlight some of the key findings described in the paper. In particular, the authors simply name the key themes and don’t really describe some of the major findings including concerns about the time needed to use the tool in primary care or how to address the next steps after risk assessment.**

Thank you for this feedback. We have now edited the results section in the abstract (page 2) to read:

*Results: Seventy-five clinicians participated, 21 from primary care and 54 from specialist genetics clinics. Participants were from England (n=37), France (n=23) and Germany (n=15). The prototype CanRisk tool was generally acceptable to most participants due to its intuitive design. Primary care were concerned about the amount of time needed to complete, interpret and communicate risk information. Clinicians from both settings were apprehensive about the impact of the CanRisk tool on their consultations and lack of opportunities to interpret risk scores before sharing with their patients.*

**2 Introduction, first sentence (minor comment): The sentence states that “…there is a need to identify people at higher risk for tailored screening and prevention”, which is an ambiguous sentence structure. It would be preferable to state it as “…there is a need to identify people at higher risk, who may benefit from tailored screening and prevention”.**

Thank you for this suggestion – we have amended the text accordingly (see page 3):

*In order to maximise opportunities to both prevent and detect cancer early, there is a need to identify people at higher risk, who may benefit from tailored screening and prevention [1-3].*

**3 Box 1: There is inconsistency in the information presented regarding the subject’s birthdate (4/2/1969), her daughter’s birthdate (3/4/1997) and the age at which she gave birth (18). Based on the birthdates, the mother was 28, not 18, when she gave birth to her daughter.**

Thank you for pointing out this error. We have now amended the age at birth in Box 1 and Box 2.

**4 Page 9, Analysis (minor comment): Clarify that “these were refined with guidance of FW” is another author.**

Thank you for making this point – we have edited the text (page 7) to read:

*The codes were drawn together into a broad set of initial themes; these were refined with guidance of the senior author (FW).*

**5 Results: Although it is recognized that this was a qualitative analysis, it would be useful to quantify how frequently some of the comments were made. For example, how many times did clinicians express concerns about the time it would take to use the tool or how many had concerns about the questions being too intrusive or upsetting. It would help the reader to know whether the concerns were reported by the many clinicians or only by one or two people. If stratified by type of practitioner, it could quickly highlight differences in perceptions and needs by clinical setting.**

Thank you for your suggestion. We have included additional information about the number of times a theme occurred in figure 4; this supplements the proportional representation of each setting already depicted. The Figure 4 legend (see page 8) now reads:

*Fig 4 – Inductive themes mapped onto Sekhon et al’s Theoretical Framework of Acceptability [12]. The total number of times the theme occurred is presented after each theme name (n=x). The yellow (primary care) and green (specialist genetics clinic) colouring shows the proportional contribution of each sample to the theme.*

**Editorial requests**

**1 Please ensure that your manuscript meets PLOS ONE's style requirements, including those for file naming.**

We have now amended the manuscript to meet the journal’s style requirements.

**2 Please include additional information regarding the survey or questionnaire used in the study and ensure that you have provided sufficient details that others could replicate the analyses. For instance, if you developed a questionnaire as part of this study and it is not under a copyright more restrictive than CC-BY, please include a copy, in both the original language and English, as Supporting Information.**

The interview schedule has been included as supplementary file 1. We have included more details on the information collected in the demographic questionnaire in the main body of the manuscript – see page 5.

*Those based in England participated face-to-face with the researcher. They completed an initial questionnaire collecting demographic data (age, gender, years in role, number of hours worked, any research qualifications) and information on their current use of computerised risk assessment tools (use of: web browsers, computer in clinic, electronic risk assessment tools, electronic tools to draw family tree).*

**3 Please provide additional details regarding participant consent. In the ethics statement in the Methods and online submission information, please ensure that you have specified what type of consent you obtained (for instance, written or verbal, and if verbal, how it was documented and witnessed). If your study included minors, state whether you obtained consent from parents or guardians**

We have now updated the ethics section to detail that participants gave written consent – see page 4). We have also edited the online submission information to reflect this.

*Following the receipt of an invitation to participate and a participant information sheet, participants gave full informed written consent.*

**4 Thank you for sending us the data set underlying the results presented in your PLOS ONE submission. We notice that some of the information included in the data set may be potentially identifying. Please ensure that the data shared are in accordance with participant consent and provide only the data that are used in this specific study. To ensure patient confidentiality, we would recommend removing any identifying information in Box 2 of the manuscript.**

The information contained in Box 1 and 2 relate to the example vignettes for a fictitious patient used in the study and do not include any data from real patients.

**5 In your Methods section, please provide additional information about the participant recruitment method and the demographic details of your participants. Please ensure you have provided sufficient details to replicate the analyses such as:**

a) **the recruitment date range (month and year)**
b) **a description of any inclusion/exclusion criteria that were applied to participant recruitment**
c) **a description of how participants were recruited**
d) **descriptions of where participants were recruited and where the research took place.**

We have included additional information in the Procedure section of the methods to address these points – please see page 5.

*The research design aimed to standardise the content and quality of data collection across countries, so we used two methods of data collection for the study (face-to-face or on-line), determined by proximity to the participants. Participants were identified through local/national/international research networks (e.g. the Clinical Research Network in the UK) and were initially contacted by email. Participants were eligible for inclusion if they worked in a specialist genetics clinic or general practice and could give informed consent. Data were collected between April 2018 and August 2018, and the study was undertaken in participant’s clinical workplaces.*

**6 We note that you have indicated that data from this study are available upon request. PLOS only allows data to be available upon request if there are legal or ethical restrictions on sharing data publicly. For information on unacceptable data access restrictions, please see** [**http://journals.plos.org/plosone/s/data-availability#loc-unacceptable-data-access-restrictions**](http://journals.plos.org/plosone/s/data-availability#loc-unacceptable-data-access-restrictions)**.**

**In your revised cover letter, please address the following prompts:**

**a) If there are ethical or legal restrictions on sharing a de-identified data set, please explain them in detail (e.g., data contain potentially identifying or sensitive patient information) and who has imposed them (e.g., an ethics committee). Please also provide contact information for a data access committee, ethics committee, or other institutional body to which data requests may be sent.**

**b) If there are no restrictions, please upload the minimal anonymized data set necessary to replicate your study findings as either Supporting Information files or to a stable, public repository and provide us with the relevant URLs, DOIs, or accession numbers. Please see** [**http://www.bmj.com/content/340/bmj.c181.long**](http://www.bmj.com/content/340/bmj.c181.long) **for guidelines on how to de-identify and prepare clinical data for publication. For a list of acceptable repositories, please see** [**http://journals.plos.org/plosone/s/data-availability#loc-recommended-repositories**](http://journals.plos.org/plosone/s/data-availability#loc-recommended-repositories)**.**

**We will update your Data Availability statement on your behalf to reflect the information you provide.**

We have included more information about this in our cover letter as requested.

**7 We note that you have a patent relating to material pertinent to this article. Please provide an amended statement of Competing Interests to declare this patent (with details including name and number), along with any other relevant declarations relating to employment, consultancy, patents, products in development or modified products etc. Please confirm that this does not alter your adherence to all PLOS ONE policies on sharing data and materials, as detailed online in our guide for authors http://journals.plos.org/plosone/s/competing-interests by including the following statement: "This does not alter our adherence to PLOS ONE policies on sharing data and materials.” If there are restrictions on sharing of data and/or materials, please state these. Please note that we cannot proceed with consideration of your article until this information has been declared.**

There is no patent relating to the tool described in this article. The CanRisk tool has recently undergone CE marking for use in the European Economic Area and is freely available. The BOADICEA mathematical algorithm is licenced to Cambridge Enterprise, and royalties may be earned by the listed inventors. We have included an amended statement of Competing Interests (page 20) to read:

*The BOADICEA model has been licensed to Cambridge Enterprise for commercialization, with the authors D.F.E., A.C.A., A.P.C., A.L. and T.C. listed as its inventors. These authors may receive royalties in the future if commercialization is realized. The other authors declare no conflicts of interest.*

**8 PLOS requires an ORCID iD for the corresponding author in Editorial Manager on papers submitted after December 6th, 2016. Please ensure that you have an ORCID iD and that it is validated in Editorial Manager.**

This has now been added and validated within the editorial manager.

**9 Please include captions for your Supporting Information files at the end of your manuscript, and update any in-text citations to match accordingly.**

These have now been included and named accordingly.
